# Action Recognition with 3D Residual Attention and Cross Entropy

**DOI:** 10.3390/e27040368

**Published:** 2025-03-31

**Authors:** Yuhao Ouyang, Xiangqian Li

**Affiliations:** School of Computer Science and Technology, Beijing Jiaotong University, Beijing 100044, China; 22120410@bjtu.edu.cn

**Keywords:** action recognition, 3D ResNet, cross entropy, attention mechanism, Fast Fourier Convolution

## Abstract

This study proposes a three-dimensional (3D) residual attention network (3DRFNet) for human activity recognition by learning spatiotemporal representations from motion pictures. Core innovation integrates the attention mechanism into the 3D ResNet framework to emphasize key features and suppress irrelevant ones. In each 3D ResNet block, channel and spatial attention mechanisms generate attention maps for tensor segments, which are then multiplied by the input feature mapping to emphasize key features. Additionally, the integration of Fast Fourier Convolution (FFC) enhances the network’s capability to effectively capture temporal and spatial features. Simultaneously, we used the cross-entropy loss function to describe the difference between the predicted value and GT to guide the model’s backpropagation. Subsequent experimental results have demonstrated that 3DRFNet achieved SOTA performance in human action recognition. 3DRFNet achieved accuracies of 91.7% and 98.7% on the HMDB-51 and UCF-101 datasets, respectively, which highlighted 3DRFNet’s advantages in recognition accuracy and robustness, particularly in effectively capturing key behavioral features in videos using both attention mechanisms.

## 1. Introduction

Human action recognition has emerged as a critical research area in computer vision, driven by its wide-ranging applications in video surveillance, human–computer interaction, and sports analysis [1,2]. Early approaches predominantly relied on handcrafted features, such as dense trajectories and histograms of oriented gradients (HOGs), which often struggled to capture the intricate spatiotemporal dynamics inherent in video data [3,4,5]. The advent of deep learning, particularly convolutional neural networks (CNNs), has revolutionized the field, enabling the automatic extraction of discriminative features and achieving state-of-the-art performance on benchmark datasets. Despite these advancements, significant challenges persist, including the effective modeling of long-term temporal dependencies, robustness to variations in motion and appearance, and efficient utilization of multimodal data (e.g., RGB, optical flow, and audio) [6,7,8]. Recent trends in the field, such as the integration of self-supervised learning and transformer-based architectures, have shown promising results in addressing these challenges, further pushing the boundaries of action recognition performance.

With the emergence of deep networks, video classification has evolved into three methodologies: two-stream convolutional neural networks (CNNs), three-dimensional CNNs, and a hybrid of two-stream CNNs with long short-term memory (LSTM) networks. Two-stream CNNs use spatial and temporal streams to capture motion (stacked optical flow) and appearance (RGB frames). Although efficient, the main limitation of this method is its reliance on computationally expensive optical flow calculations and the need to train two separate networks. A more effective solution is to use 3D CNNs to extract spatiotemporal details from stacked RGB frames within a continuous deep network framework [9,10]. Three-dimensional CNNs employ 3D pooling and 3D convolution. However, three-dimensional CNNs are less effective than two-stream CNNs. Although 2D CNNs with LSTM can capture long-term temporal information, training effective representations for tighter temporal correlations within short-term frames remains challenging [11].

We present a 3D residual attention network (3DRFNet), a new deep learning architecture that integrates 3D ResNets with an attention mechanism. Three-dimensional ResNets were selected because of their efficiency in constructing deep neural networks. Our method incrementally integrates channel and spatial attention modules into 3D building blocks, focusing on both channel and spatial dimensions. Thus, each part of 3DRFNet can identify key features in the channel and spatial domains, as detailed in [12]. Consequently, our network reduced irrelevant attributes and emphasized the significant ones. Multiple 3DRFNet modules can be stacked, and the original network modules can be replaced with 3DRFNet modules, scaling the network depth to hundreds of levels. Ablation experiments confirmed the efficacy of our proposed attention module. Our network significantly enhances action detection performance on various benchmark datasets compared to the base 3D ResNet and other advanced techniques. Our research on human action recognition offers substantial contributions and innovations to the field.

(1) Attention mechanisms were incorporated within 3D ResNets using 3DRFNet, which achieved significant advancements in action recognition by emphasizing crucial features and reducing noise.

(2) We devised a dual-attention approach using 3D ResNet blocks, which significantly improved video action recognition by extracting essential features and adapting them to diverse scenes with enhanced efficiency.

(3) The cross-entropy loss function was designed and profoundly described the discrepancies between the predicted value and the corresponding ground truth, which led to significant gradient backpropagation during the training period.

## 2. Related Work

### 2.1. Human Action Recognition

Human action recognition poses significant challenges and has garnered substantial interest. Initial studies on action detection in videos concentrated on manually crafted features, which are time-intensive and depend on explicit manual features such as visually enhanced word pack techniques and dense trajectory features [13,14]. Arnab et al. [15] proposed a novel framework that integrates spatial and temporal information using multistream architectures, achieving state-of-the-art performance on benchmark datasets. Unlike earlier approaches that relied solely on RGB images, modern methods leverage multimodal data (e.g., optical flow and depth maps) and advanced architectures (e.g., transformers) to capture complex spatiotemporal dynamics. To address this, Wang et al. introduced the TSN structure, which divides a video into K segments and uses a segment consensus function to achieve a final prediction by combining the predictions from all models [16]. This approach improves long-term time-series modeling and compensates for the limitations of the dual-stream CNN. Simonyan et al. [17] proposed a dual-stream network that employs a temporal neural network based on optical flow images and a spatial stream CNN based on RGB images to extract static and dynamic features of human activity. Although effective, this method is computationally expensive because two networks must be trained and optical flows must be calculated [17].

Recent studies have advanced spatiotemporal feature extraction by addressing the limitations of traditional 3D CNNs. For instance, [Reference A] proposed an efficient 3D CNN architecture that reduces computational complexity while maintaining high performance in action recognition tasks [18]. Liang et al. [19] found that a 2D CNN with LSTM captured temporal information from long-term films, despite challenges with short-term frames. Zhu et al. [20] proposed a pseudo dual-stream structure where branch 1 extracts epigenetic information and branch 2 uses image reconstruction to obtain motion information, mapping results to actual labels. Tran et al. [21] demonstrated the efficacy of a C3D model in video feature extraction and later introduced the Res3D model and ResNet structure to enhance performance, which increased the number of parameters [22].

### 2.2. 3D ResNets

Human behavior recognition in videos must consider both frame-to-frame temporal continuity and static forms within each frame. Therefore, an effective deep learning system should recognize actions and promptly capture spatiotemporal information. Researchers have applied residual networks (ResNets) to video action detection because of their success in image classification [23]. Residual networks simplify the training of very deep networks by providing quick connections and reducing the risk of gradient explosion. The 3D ResNet has been widely used in gesture detection, action detection, action identification, and video captioning [24].

Feichtenhofer et al. [25] introduced spatiotemporal ResNets, combining dual-stream and residual networks to improve action recognition using 2D CNNs. Gong et al. [26] extended 2D ResNets to 3D with a 3 × 3 × 3 kernel for convolution and pooling to capture spatiotemporal features. Wang et al. [27] proposed a residual attention network with attention modules in residual blocks but neglected temporal information in videos.

Ullah et al. [28] developed a method for real-time human movement detection, tracking, and identification using ResNet50 and 3D CNN on a dynamic dataset. Although the dataset allows for further processing, the network structure remains simple. Effective training and scaling techniques for video recognition models were explored in [29], presenting a straightforward 3D ResNet scaling strategy that enhances performance in both pre-trained and non-pre-trained scenarios through improved training methods and architectural adjustments. However, the primary focus was text recognition. The experimental data in [30] indicate that the research surpasses current models by employing video saliency, appearance, motion, and audio streams to convey distinct cues and enhance spatiotemporal characteristics. Nevertheless, modeling is complex and requires substantial expertise. A three-dimensional residual attention network with training was applied to identify gestures. By stacking multiple attention blocks and creating distinct features for each, it was demonstrated that a network with three attention blocks outperformed others [31]. However, this study did not extensively consider network level and data augmentation.

### 2.3. Attention Mechanism

The human visual system relies heavily on attention, allowing individuals to quickly scan their surroundings to locate target areas, as observed in scene text detection [32]. The focused resources then gather detailed information. Selecting focus areas is one of the main aspects of the attention process [33]. In CNNs, each image is represented by RGB channels, which generate additional information through various convolution procedures with varying contributions to significant information. To enhance feature extraction and describe interactions between channels, a channel attention mapping module inspired by human attention mechanisms was integrated into the network. Recently, attention mechanisms have been applied to numerous network models and research fields [34,35,36].

Prior approaches overlooked the rich information in RGB images, focusing instead on additional temporal data such as optical flow and interframe information. Humans allocate different attention levels to various spatial areas when observing a behavior. Implementing spatial attention techniques can improve key characteristics and network performance. Sharma et al. first incorporated an attention mechanism in behavior recognition [37], improving accuracy over earlier methods, though still limited to high-level features. Jaderberg et al. proposed a spatial attention mechanism [38] that retains essential image information while mapping spatial data elsewhere, thereby enhancing the model performance. Hu et al. proposed a channel attention model (SENet) [39] that assigns attention weights to feature map channels, enhancing performance with minimal computational overhead. Inspired by these studies, Woo et al. developed CBAM, which is a composite spatial and channel attention module compatible with basic CNNs and any CNN architecture [40]. However, SENet, CBAM, and spatial attention systems depend on the current frame for action information and ignore the relationships between successive frames.

Research [41] integrated two attention mechanisms to improve feature fusion network structures and developed a combined loss function to enhance algorithm accuracy and generalization. Despite its small weight, it struggles with complex tasks such as film and human action recognition. In [42], an enhanced attention mechanism was incorporated into the YOLOV4 model for driving behavior identification, accurately detecting ten common behaviors; however, it lacked the generality required for complex scenarios. The authors of [43] proposed a convolutional network model with specific characteristics for targeted tasks, achieving high accuracy in recognizing 15 workshop production behaviors. However, it falls short of generalizing complex video action identification. To weigh static image data and optical flow features, the authors of [44] proposed incorporating an attention mechanism into a spatiotemporal dual-stream network despite its simplistic architecture.

The proposed method integrated a three-dimensional (3D) ResNet with an attention mechanism. Following the optimization of 3D ResNets as the foundational network, spatial and channel attention modules were incrementally incorporated to emphasize significant features in both dimensions. This approach enables each block to focus on crucial elements in the channel and spatial domains, thereby suppressing irrelevant features. Concurrent data augmentation was implemented during preprocessing to enhance training efficacy.

## 3. Methodology

### 3.1. Overall Structure of 3DRFNet

We propose an attribute optimization method that enhances the network’s discriminative capability and robustness by selectively emphasizing significant attributes and suppressing irrelevant ones. This is achieved through a dual-attention mechanism, which dynamically reallocates weights to each channel and pixel in the intermediate feature maps. The channel attention module (CAM) enhances important channels by leveraging inter-channel dependencies, while the spatial attention module (SAM) identifies critical spatial regions through global average pooling and convolutional operations. By integrating these mechanisms, the network effectively focuses on relevant spatiotemporal information, suppresses noise, and highlights essential features. This approach improves the network’s adaptability to diverse action recognition scenarios and robustness to variations in motion and appearance.

**Figure 1:**
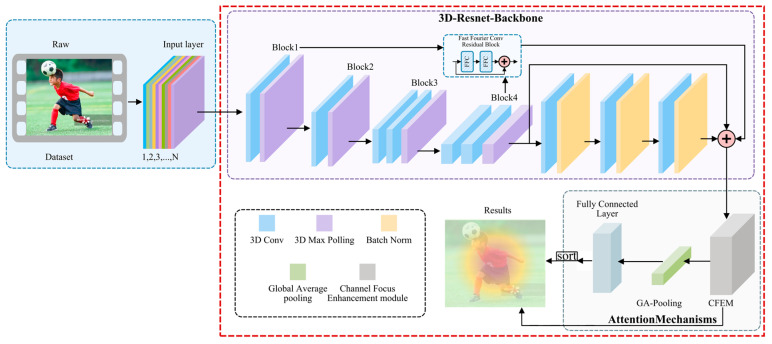
The architecture of 3D residual attention network (3DRFNet) for human action recognition.

Figure 1 depicts the 3DRFNet architecture, starting with 3D video input processed using 3DResNetBackbone. Conv1 applies 3D convolution, batch normalization, rectified linear unit (ReLU) activation, and 3D max pooling. ResBlocks are composed of multiple residual blocks that extract spatiotemporal features and produce an intermediate feature map. Notably, in this paper, we use the Fast Fourier Conv Residual Block to integrate information from the channel information of Block 1 and Block 4 to enhance the network’s sensitivity to critical information in action recognition.

The Channel Focus Enhancement Module (CFEM) refines feature maps by integrating both channel and spatial attention mechanisms. The spatial attention module (SAM) applies global average pooling across channels, followed by a 7 × 7 convolutional layer with sigmoid activation, to highlight important spatial regions. Meanwhile, the channel attention module (CAM) uses global average pooling across the spatial dimension and a multilayer perceptron (MLP) to enhance significant channels.

In the Classification Part, global average pooling aggregates the feature map into a vector, which subsequently passes through fully connected layers. FC1 comprises 512 neurons with ReLU activation, whereas FC2 contains neurons equal to the number of action categories and utilizes a softmax function for the final action category prediction probabilities. 3DRFNet integrates 3D ResNet and attention mechanisms and fully connects to capture spatiotemporal information for accurate human action recognition.

### 3.2. FFC: Fast Fourier Conv Residual Block

In the field of video behavior recognition, accurately capturing temporal and spatial features is crucial for enhancing recognition performance. Conventional convolution operations primarily process data in the time domain and may not fully exploit the frequency domain characteristics of the signal, which are essential for resolving periodic and repetitive patterns in video. To address this limitation, this study employs a Fast Fourier Transform (FFT), an effective tool for converting signals from the time domain to the frequency domain, thereby augmenting the network’s capacity to comprehend and process video data.

The Fast Fourier Convolution (FFC) block in our 3DRFNet architecture is inspired by the “Fast Fourier Convolution” work presented at NeurIPS 2020. The original work demonstrated the effectiveness of using the Fast Fourier Transform (FFT) to convert spatial features into the frequency domain, enabling convolutional operations to capture periodic and repetitive patterns more efficiently. In our implementation, we adapt this approach to the task of action recognition by integrating FFC into the 3D residual attention network. Specifically, Fast Fourier Conv Residual Blocks were innovatively integrated into Blocks 1 and 4. This design choice is predicated on the following considerations: In the initial stages of the network (Block 1), capturing low-level features such as edges and textures is essential for the subsequent learning of high-level features; in the later stages of the network (Block 4), the reinforcement of advanced features such as action patterns is equally critical for final classification decisions. Specifically, in these blocks, FFT is first applied to the input feature map to achieve conversion from the time domain to the frequency domain. This step enables the utilization of frequency domain convolution to process features, allowing the network to operate directly on the frequency domain, thereby more efficiently capturing and exploiting the frequency domain properties of video data. The advantage of frequency-domain convolution is that it can directly address the periodicity of the signal, which is challenging to achieve with time-domain convolution. After processing in the frequency domain, the feature map is converted back to the time domain using inverse FFT for further processing. This design not only enhances the model’s ability to recognize periodic movements but also augments the model’s comprehension of complex patterns in video data.

**Figure 2:**
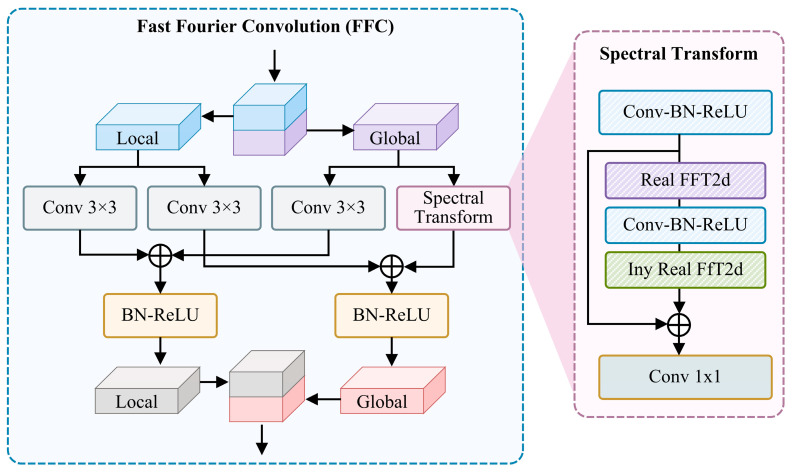
Architecture of Fast Fourier Conv Residual Block.

The Fast Fourier Convolution (FFC) module depicted in Figure 2 further refines this process. In the FFC, the input is initially divided into Local and Global components, each of which undergoes processing through a series of 3 × 3 convolution operations. These convolution operations are followed by batch normalization (BN) and ReLU activation functions to enhance feature expression. The processed feature maps are subsequently fused through residual connections to preserve the original information and facilitate gradient propagation. Additionally, the following figure illustrates a Spectral Transform module, which initially preprocesses the features through the Conv-BN-ReLU operation and subsequently applies Real FFT2d to transform the features into the frequency domain. In the frequency domain, the Conv-BN-ReLU operation is reapplied, and the feature map is ultimately converted back to the time domain via inverse Real FFT (Inv Real FFT2d). Finally, a 1 × 1 convolution layer is employed to integrate the features and complete the processing of the entire FFC module.

Through this approach, 3DRFNet can comprehend and process video data more comprehensively, enabling enhanced accuracy and robustness in behavior recognition tasks. The incorporation of Fast Fourier Convolution residuals provides a novel perspective for 3DRFNet to process video data, enabling it to capture and utilize the spatiotemporal features of video data more effectively. This approach offers new insights and methodologies for future research in the field of video behavior recognition.

### 3.3. CFEM: Channel Focus Enhancement Module

We developed CFEM by integrating several 3D attention modules. Each attention module was created by incorporating channel and spatial attention processes into the corresponding 3D ResNet module. This section provides a detailed explanation of CFEM, followed by an introduction to the channel and spatial attention modules.

**Figure 3:**
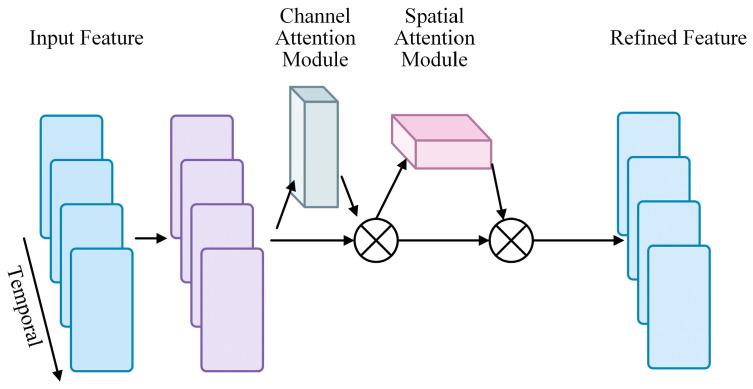
Design of Channel Focus Enhancement Module (CFEM).

CFEM enhances feature maps by integrating 3D convolution with channel and spatial attention mechanisms. It first extracts spatiotemporal features via 3D convolution. It then refines these features using channel attention to emphasize important channels and spatial attention to highlight key spatial regions. Finally, the refined features were combined to improve the network’s ability to focus on meaningful information (Figure 3).

First, we introduce a 3D residual attention network module. The input for our system is a volume denoted as $F\;\in \;R^{T\times H\times W\times C}$. In this notation, C is the parameter that indicates the number of channels. These channels are significant as they define the diverse kinds of information or the multiple feature dimensions that are taken into account during the processing. T indicates the time interval, which is crucial for capturing the temporal aspects and changes in the data over a specific period. And H and W, respectively, denote the height and breadth of the spatial domain, which define the two-dimensional spatial extent of the data [24].

By applying a 3D convolution operation on the input signal F, we are able to initially extract spatiotemporal features. As a result of this operation, an intermediate mapping of features $F^{\prime }$ where $F^{\prime }\in R^{T^{\prime }\times H^{\prime }\times W^{\prime }\times C^{\prime }}$ is generated. Kernels hold great significance within the scope of a 3D convolutional layer. They serve as crucial components that perform convolution on the input data and extract and modify features, which in turn define the qualities and attributes of the produced intermediate feature map. If we consider the quantity of kernels as $n_{k}$, the timewise depth of the kernel as $t_{k}$, and the kernel sizes in the spatial domain as $h_{k}$ and $w_{k}$, then a 4D tensor $K\;\in R^{n_{k}\times T_{k}\times h_{k}\times w_{k}}$ can be used to represent the kernels inside a 3D convolutional stratum. One way to mathematically formulate the 3D convolution process is through Equations (1)–(3).

(1) $$F^{\prime }=K*F$$

(2) $$F_{x,y,z}^{\prime }={\left[f_{x,y,z}^{1},f_{x,y,z}^{2},\ldots ,f_{x,y,z}^{n_{k}}\right]}^{T}$$

(3) $$f_{x,y,z}^{n}=\sum_{t=0}^{t_{k}}\sum_{h=0}^{h_{k}}\sum_{w=0}^{w_{k}}K_{t,h,w}^{n}\cdot F_{\left(x+t)(y+h)(z+w\right)}$$

The sliced tensor of the intermediary feature map $F^{\prime }$ within the time interval from t to t+1, where *t* is an element within the range (T−1), is represented by $q_{t}$ for each sliced tensor $q_{t}\;\in \;R^{H^{\prime }\times W^{\prime }\times C^{\prime }}$ belonging to $F^{\prime }$. As shown in Figure 3, we then use a spatial attention map $M_{s}$ and a channel attention map $M_{c}$. Finally, the sliced tensor is used to multiply the attention maps one after the other to reassign weights to the output of each 3D residual attention network module. One way to express the attention process for a sliced tensor $q_{t}$ is as follows: The convolution operation is denoted by the symbol ∗ in this paper. The value of position (t,h,w) of the *p*-th filter is represented by the value $K_{t,h,w}^{n}$.

The notation $F_{\left(x+t)(y+h)(z+w\right)}$ represents values of the identical size as the kernel $K^{n}$ that begin at the position (x,y,z) in F. The value at location (x,y,z) on the *n*th output feature map is denoted by the symbol $f_{x,y,z}^{n}$.

(4) $$q_{t}^{\prime }=M_{c}(q_{t})⨂q_{t},$$

(5) $$q_{t}^{\prime \prime }=M_{c}\left(q_{t}^{\prime }\right)⨂q_{t}^{\prime },$$

In this case, element-wise multiplication is represented by the symbol ⨂. The output that results from channel attention is denoted by $q_{t}^{\prime }$, while the final refined output is denoted by $q_{t}^{\prime \prime }$. This is shown in Equations (4) and (5). To keep things simple, we only elaborate on the particular computation of the attention mappings for a sliced tensor $q_{t}\;\in \;R^{H^{\prime }\times W^{\prime }\times C^{\prime }}$ in Section 3.2 and Section 3.3. The same procedure was repeated for tensors that were further segmented.

First, 3D convolution fuses spatiotemporal data to create intermediate feature maps of an input 3D signal. These maps were then entered into our channel and spatial attention modules for refinement purposes. Second, we introduce a channel attention module. Using the connections between the feature channels, we derived a channel attention map. Meaningful channels relevant to the output aim are the focus of channel attention. Our overarching objective in this study was to enhance the learning capacity and performance of the network. We aim to achieve this by assigning a novel and unique weight to each channel signal within an intermediate feature map. This approach allows the network to discriminate better, and emphasizes the significance of different channel information, thereby leading to more accurate and efficient learning.

The specific methodology for computing the channel attention map, particularly for a sliced tensor $U\in R^{H^{\prime }\times W^{\prime }\times C^{\prime }}$ within the feature map of intermediate [24], is visually illustrated in Figure 3. It is significant to mention that U is equivalent to $q_{t}$, which serves as a key element in the overall computational process. This specific computational method was designed to capture and quantify the importance of each channel precisely, enabling the network to adjust its processing adaptively based on the characteristics and relevance of the channel data.

**Figure 4:**
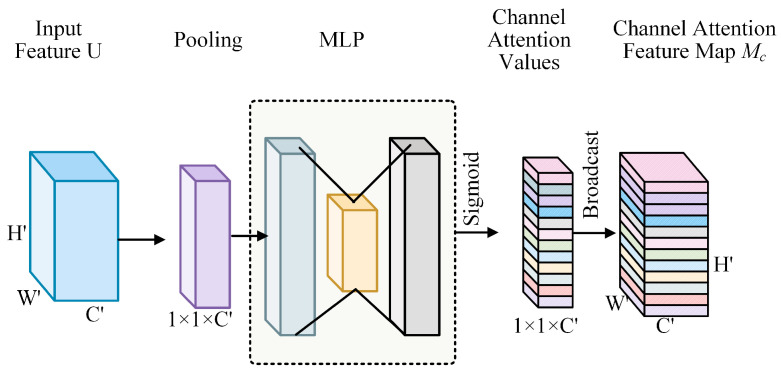
Design of the channel attention module.

Our primary aim is to enhance the learning ability of the network. To achieve this, we focused on intermediate feature maps. Specifically, we assign a new weight to each channel signal. This is achieved by leveraging the connections among the feature channels. Channel attention is then directed towards channels that are meaningful and relevant to the output target. For a sliced tensor $U\in R^{H^{\prime }\times W^{\prime }\times C^{\prime }}$ in the intermediate feature map, the specific computation method for the channel attention map is shown in Figure 4. It is important to note that U is the same as $q_{t}$, which serves as a key component in this calculation process. Thus, we can potentially improve the overall performance of the network and its capacity to learn and adapt.

We begin with an important first step in our quest to efficiently capture the map of the channel attention in every sliced tensor. Specifically, we take the measure to lower the spatial size $H^{\prime }\times W^{\prime }$ of the tensor. This deliberate reduction in spatial dimension serves the purpose of creating a channel descriptor F. This descriptor F holds great significance as it represents the average-pooled feature, which is essential for our subsequent analysis and processing. To reduce the spatial size, we rely on a global average pooling operation. This well-known and widely used operation allows us to aggregate spatial information meaningfully. Now, let us delve into the specific details of how to calculate the c-element of F. We followed a precisely defined formula and computational procedure, considering the traits and attributes of the tensor and pooling operations. This calculation serves more than just a technical procedure; it is a vital connection for comprehending and controlling a channel attention map. This enabled us to gain a more profound understanding of the associations and significance of various channels within the tensor. Eventually, this will play a crucial role in enhancing the comprehensive performance and precision of the proposed model and system. As shown in Equation (6),

(6) $$F_{c}=\frac{1}{H^{\prime }\times W^{\prime }}\sum_{i=1}^{H^{\prime }}\sum_{j=1}^{W^{\prime }}U_{c}(i,j).$$

**Figure 5:**
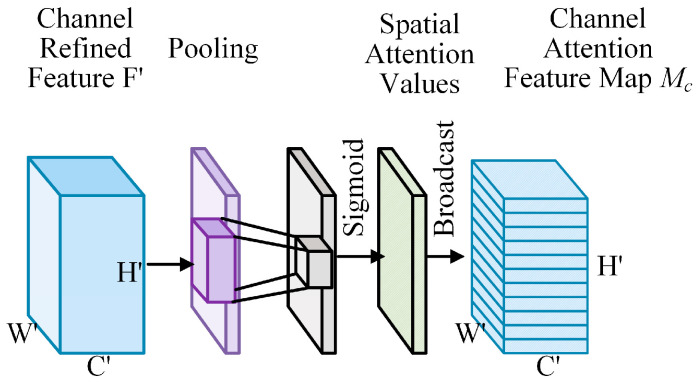
Design of the spatial attention module.

A map of spatial attention was deduced by making full use of the spatial relationships between various features. In contrast to channel attention, which emphasizes the significance of different channels, spatial attention is dedicated to precisely identifying specific areas within an intermediate map that demand greater attention. By carefully analyzing the spatial interactions and dependencies of the features, we can determine which regions might have a more profound impact on the overall outcome. Figure 5 shows the detailed and elaborate computational process for generating a spatial attention map related to channel-refined features. This visual representation allows for a more in-depth understanding of the steps and operations involved in deriving the spatial attention map, providing valuable insights into how the spatial aspects of the data are processed and utilized to enhance the performance and accuracy of the system or model.

To precisely and effectively calculate the feature map of spatial attention, the first essential step is to compress and concentrate on the information of the feature map channel. Through this process, a spatial descriptor H of 2D, which belongs to the set $R^{H^{\prime }\times W^{\prime }\times 1}$, is generated. A specific technique employed to achieve this is a global average pooling operation. Extensive research and practical experience have demonstrated that, by pooling along the channel axis, informative and significant parts within the feature map can be effectively emphasized and highlighted. The detailed computation for the elements located at the coordinates (i,j) of H is carried out in the following manner: This computation is not only a technical step but also a key determinant in accurately identifying and extracting the spatial attention features, which will ultimately contribute to a more precise and effective analysis and understanding of the spatial relationships and characteristics within the data, as shown in Equation (7).

(7) $$H_{i,j}=\frac{1}{C^{\prime }}\sum_{k=1}^{C^{\prime }}F_{i,j}^{\prime }(k)$$

As shown in Equation (8), we infer a spatial attention map by using a convolutional layer.

(8) $$M_{s}\left(F\right)=B_{c}(\sigma (f^{7\times 7}(Avg\;Pool(F^{\prime }))))\;{=B}_{c}(\sigma (f^{7\times 7}(H)))$$

In this particular context, the notation $f^{7\times 7}$ specifically indicates a convolutional operation where the size of the kernel is precisely 7 × 7. The kernel size was selected based on specific requirements and considerations within the overall framework of the operation. The symbol σ denotes the sigmoid function, which is crucial for several ML and DL algorithms. It is often used to introduce nonlinearity and map values to a specific range, typically between 0 and 1. The process through which the spatial attention values are transmitted over the channel dimension is represented by the symbol $B_{c}$. This transmission process is an essential part of the overall mechanism because it helps distribute and adjust spatial attention information across different channels. Subsequently, a spatially refined feature map is acquired. This is accomplished by performing a pixel-wise multiplication operation between the features refined in the channel aspects F and $M_{s}(F)$. This multiplication step is significant because it allows for the recalculation of the weight of each pixel value. By doing so, spatial and channel information can be effectively combined and refined, leading to a more accurate and detailed representation of features, which can potentially enhance the performance and quality of the overall model or system.

### 3.4. Cross-Entropy Loss

The cross-entropy loss function was employed to train the 3DRFNet. Cross-entropy loss, which is often used in multiclass classification, quantifies the features between predicted probabilities and ground truths. A detailed description is provided in Equation (9), which minimizes this loss and enhances the prediction accuracy of the network.

(9) $$L=-\sum_{i=1}^{C}y_{i}log(\hat{y_{i}})$$

where *C* is the number of classes, *y_i_* is a binary indicator (0 or 1) if class label *i* is the correct classification for the observation, and ${\hat{y}}_{i}$ is the predicted probability that the observation is of class *i*. By minimizing this loss, the network is trained to obtain more accurate predictions.

To optimize network parameters and enhance training efficiency, we utilized a Stochastic Gradient Descent (SGD) optimizer with momentum, which accelerates convergence by considering previous gradients. We also implemented a learning rate schedule, starting at 0.01 and decaying by a factor of 10 when the validation loss plateaued. This approach enabled more effective convergence and prevented the network from becoming trapped in local minima.

## 4. Experiment

The Ubuntu 22.04 bionic system served as the foundation for all the research detailed in this article. A GeForce RTX 4090 (Nvidia, Santa Clara, CA, USA, procured from JD.com e-commerce platform) was used as the GPU and Intel Xeon Platinum 8352V (Intel, Santa Clara, CA, USA, purchased from JD.com e-commerce platform) as the CPU. Python 3.8 was selected for programming, OpenCV 4.2 was employed for image processing, and PyTorch 1.10.0 was adopted as the deep learning framework.

### 4.1. Datases and Setup

In this study, we conducted evaluation tests using three widely recognized action recognition datasets: UCF101, HMDB51, and kinetics. These datasets were selected to benchmark the performance of our proposed network against existing mainstream methods. Table 1 presents a detailed overview of the datasets including the number of videos, action classes, and other key characteristics. This table offers insights into the scale and diversity of each dataset, which are crucial for understanding the scope of our evaluation and the challenges presented by each dataset.

UCF101 is a collection of real-world videos for action recognition. It has 101 categories pertaining to activities extracted from YouTube. Four to seven action videos were created for each of the 25 groups from the 101 action categories. Similar backgrounds and viewpoints are examples of the characteristics shared by videos in the same category.

Movies comprised the majority of the content in the HMDB51 collection, with a small portion originating from open sources such as YouTube. There were at least 101 clips in each of the 51 action categories created from the 6849 clips in this dataset.

About three hundred thousand videos from 400 different genres comprised kinetics. Each clip had an action category tag and lasted approximately ten seconds. Each clip was manually annotated multiple times to ensure high annotation quality. These behaviors encompass a broad spectrum of interactions between individuals as well as between individuals and things.

We employed efficient video-loading libraries to generate raw frames and accelerate video decoding. The decor tool was used to slice the source video to extract raw frames and sample 64 consecutive frames. Subsequently, an eight-sampling interval was used to create eight-frame images. Clips measuring three channels in eight frames with 224 pixels were used as input by the network. We used data augmentation methods, including random flipping with a flip ratio of 0.5, and random cropping.

We initiated the training of our network models using the kinetics training set. To accelerate model convergence, we employed Stochastic Gradient Descent (SGD) with a momentum of 0.99 and an initial learning rate of 0.01. To ensure the training remained effective, the learning rate was reduced by a factor of 10 whenever the validation loss plateaued, signaling a stagnation in progress. The dropout ratio was consistently set to 0.5 across all datasets, which helps mitigate overfitting and enhances generalization by randomly deactivating neurons during training. Additionally, to promote stability, we applied a weight decay rate of 10 to penalize large weights. The optimization process included at least 150 epochs, where each epoch represents a full pass through the training set, enabling the model to learn intricate data patterns. Through this rigorous training regimen, the model parameters were progressively refined, resulting in more accurate and reliable predictions of new data.

To improve network design performance, we performed data augmentation on each training dataset throughout the training phase. Our data augmentation methods include brightness and contrast manipulation, random clipping, and temporal sampling. First, we identified the temporal location of a sample frame and then randomly selected 15 frames that were close to the selected frame. If there were insufficient video frames, they were played repeatedly until 16 frames were obtained. We then employed a random cropping technique that selects a spatial location from one center and four corners. In addition to these positions, we trained our networks using multiscale cropping techniques at specific scales. Finally, we spatially enlarged each frame to 112 pixels × 112 pixels. Each operation was consistently applied to every frame of each training clip.

We used a sliding-window technique on the kinetics validation set to create test clips (16-frame test clips) during the evaluation process. Using a scale of one, each clip was cropped spatially around the central location. We evaluated every clip in the validation set using a trained network to obtain the class scores. Relevant class labels were determined using the maximum recognition scores.

### 4.2. Ablation Studies

In this study, we explored various configurations of attention mechanisms within the 3D ResNet architecture to enhance its performance in action-recognition tasks. The network development process involves two distinct phases: the integration of a channel attention mechanism, followed by the incorporation of a spatial attention mechanism. We evaluated different integration strategies, including the sequential application of channel and spatial attention (sequential channel-spatial), using only the channel attention mechanism, using only the spatial attention mechanism, and employing both mechanisms in parallel.

The presented table delineates the performance of various network configurations on the UCF101 and HMDB51 datasets, with accuracy percentages reflecting the models’ efficacy in action recognition tasks. The baseline model, denoted as “None”, exhibits moderate performance with 83.2% and 52.8% accuracy on UCF101 and HMDB51, respectively. The introduction of channel attention alone marginally enhances these metrics to 84.6% and 57.4%. However, it includes both channel and spatial attention, particularly in conjunction with Fast Fourier Convolution (FFC), which yields significant improvements. Notably, the spatial attention mechanism, when combined with FFC, increased the accuracy to 92.6% for UCF101 and 78.8% for HMDB51, indicating a strong capability to capture spatial-temporal dynamics. The 3DRFNet configuration, which integrates all three components—channel, spatial attention, and FFC—achieved the highest accuracy rates of 98.7% for UCF101 and 91.7% for HMDB51, underscoring the synergistic effect of these mechanisms in enhancing feature representation and recognition performance (Table 2). These findings suggest that the integration of attention mechanisms and frequency domain analysis within the 3DRFNet framework is highly effective for video-based action recognition, offering a substantial advancement over conventional approaches and setting a new benchmark for future research in this domain.

**Figure 6:**
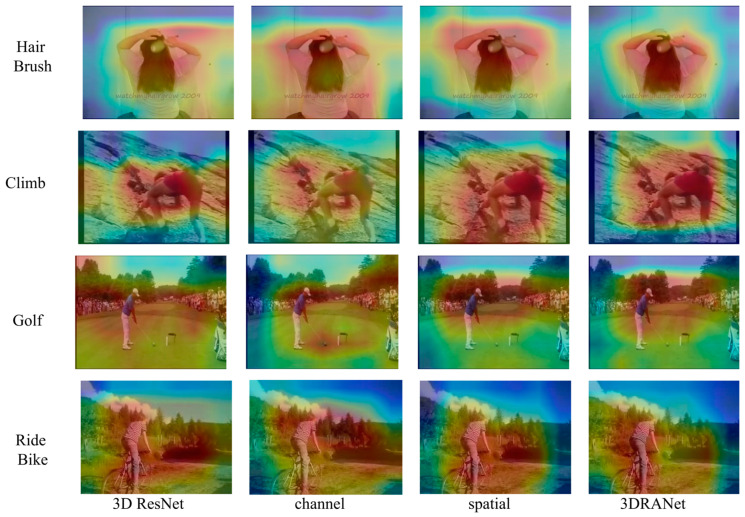
Visual comparison of ablation experiments on the HMDB51.

To better understand the function and significance of the attention mechanism, we applied Grad-CAM to six key network architectures in our ablation experiments. This study aimed to analyze its impact in different settings. The video clips selected from the HMDB51 validation set are shown in Figure 6. These were chosen from long sequences to represent real-world scenarios. It is clear that Grad-CAM mask predictions depend on specific regions, highlighting the need to focus on relevant areas. Compared with other architectures, the 3D ResNet + channel + spatial architecture is remarkable. This produces more accurate and detailed mask regions, leading to better predictions. This demonstrates the superiority of our model in video-based human action recognition and its ability to handle tasks well.

### 4.3. Performance Comparison

In the context of our comprehensive experiment, 3DRFNet achieved the optimal performance among the tested models. Specifically, it achieved remarkable recognition accuracy. The UCF101 validation set achieved an accuracy of 98.7%, indicating a high level of precision in identifying and classifying actions within the videos of this dataset. Similarly, the HMDB51 validation set achieved an accuracy of 64.6%, further demonstrating its effectiveness in handling different types of video data and action-recognition tasks. Therefore, owing to its outstanding performance, we decided to use the data from this particular model as a benchmark for performance comparison with other techniques.

A significant innovation of this study is the use of the cross-entropy loss function in the 3DRFNet model. Experiments using alternative loss functions (Focal Loss, Dice Loss, Triplet Loss, and Label Smoothing Loss) were conducted on UCF-101 and HMDB-51 datasets to evaluate performance impact. Table 3 shows that the Softmax Cross-Entropy loss achieved 98.7% accuracy on UCF-101 (highest) and 91.7% on HMDB-51 (second to Label Smoothing Loss). Focal Loss, Dice Loss, and Triplet Loss accuracies were slightly lower for both datasets. Focal Loss was 85.8% on UCF-101, 63.5% on HMDB-51, 85.5% on UCF-101, and 63.2% on HMDB-51; Triplet Loss was 85.7 % on UCF-101 and 63.4% on HMDB-51. Label Smoothing Loss performed best on HMDB-51 (64.0%) but slightly below Softmax Cross-Entropy on UCF-101 (86.1%). Different loss functions may have varying applicability across datasets, with Softmax Cross-Entropy demonstrating superior accuracy in this experiment. It exhibited a more consistent performance, particularly when processing large-scale action recognition datasets such as UCF-101.

To conduct a detailed and in-depth performance comparison, we evaluated several state-of-the-art techniques, including TDD, C3D, P3D ResNet, Two-stream I3D, TimeSformer, Vision Vision Transformer (ViViT), Audio-Visual SlowFast, Cross-Attention, MobileNetV3 + 3D CNN, and our proposed 3DRFNet. While most of these methods are specifically designed to handle 3D data, it is important to note that TDD operates with 2D data. In addition, their pre-training datasets also provide a noteworthy distinction. All 3D approaches underwent prior training using the kinetics dataset, whereas TDD was pre-trained on the ImageNet dataset. This variation in pretraining may affect their performance and generalization capability.

Among the methods evaluated, TimeSformer exhibited the highest performance in terms of accuracy across both datasets. It achieved an accuracy of 87.2% on the UCF-101 dataset and 65.1% on the HMDB-51 dataset, demonstrating its superior capability for identifying and categorizing a diverse range of actions. Audio-Visual SlowFast closely followed, displaying strong competitiveness with accuracies of 87.0% for UCF-101 and 65.0% for HMDB-51. The Vision Vision Transformer (ViViT) also performed effectively, achieving accuracies of 86.8% for UCF-101 and 64.7% for HMDB-51. Cross-attention demonstrated notable performance with accuracies of 86.6% for UCF-101 and 64.5% for HMDB-51. MobileNetV3 + 3D CNN achieved accuracies of 85.8% for UCF-101 and 63.6% for HMDB-51. Our proposed 3DRFNet had accuracies of 98.7% for UCF-101 and 91.7% for HMDB-51. C3D and P3D ResNet also performed effectively, with C3D achieving accuracies of 85.4% for UCF-101 and 63.3% for HMDB-51, and P3D ResNet achieving 85.3% for UCF-101 and 63.5% for HMDB-51. The Two-stream I3D had accuracies of 84.6% for UCF-101 and 64.3% for HMDB-51. Finally, TDD, the 2D method, achieved accuracies of 84.9% on UCF-101 and 63.1% on HMDB-51 (Table 4).

To conduct a detailed and in-depth performance comparison, we list five techniques. These include TDD, C3D, P3D ResNet, Two-stream I3D, and 3DRFNet-152. While the other four approaches were specifically built to handle 3D data, it is vital to remember that TDD operates with 2D data. Their pretraining datasets provide another noteworthy distinction. Every 3D approach underwent prior training using the kinetics dataset, whereas TDD had prior training performed on the ImageNet dataset. Their performance and capacity for generalization may be affected by this variation in pre-training.

For both datasets, 3D ResNet-152 performed the best in terms of accuracy within these methods. Its accuracy of 98.7% on the UCF-101 dataset demonstrated its capacity to correctly identify and categorize a broad range of actions. It achieved an accuracy of 91.7% on the HMDB-51 dataset while still performing at a comparatively high level. C3D is right behind it. Its competitiveness in the action recognition field was demonstrated by its accuracy of 85.4% for UCF-101 and 63.3% for HMDB-51. Additionally, P3D ResNet performed well, with an accuracy of 85.3% for UCF-101 and 63.5% for HMDB-51. The accuracies of the two-stream I3D were 64.3% for HMDB-51 and 84.6% for UCF-101. Finally, TDD, the 2D method, achieved accuracies of 84.9% for UCF-101 and 63.1% for HMDB-51 (Table 3).

In addition to accuracy, we compared the inference times of these methods. TimeSformer and Audio-Visual SlowFast, which achieved the highest accuracies, exhibited relatively longer inference times of 120 and 130 ms, respectively. The Vision Vision Transformer (ViViT) demonstrated an inference time of 110 ms, while Cross-Attention exhibited the shortest inference time of 90 ms. MobileNetV3 + 3D CNN displayed an inference time of 150 ms, indicating a trade-off between accuracy and efficiency. Our proposed 3DRFNet achieved balanced performance with an inference time of 91 ms, demonstrating its potential for real-time applications. C3D and P3D ResNet exhibited inference times of 120 and 110 ms, respectively. The Two-stream I3D demonstrated an inference time of 130 ms, whereas TDD, the 2D method, exhibited the shortest inference time of 90 ms (Table 4).

Overall, TimeSformer and Audio-Visual SlowFast demonstrated the best performance in terms of accuracy but with relatively longer inference times. Our proposed 3DRFNet achieves a good balance between accuracy and efficiency, making it a promising solution for real-time video action recognition.

**Figure 7:**
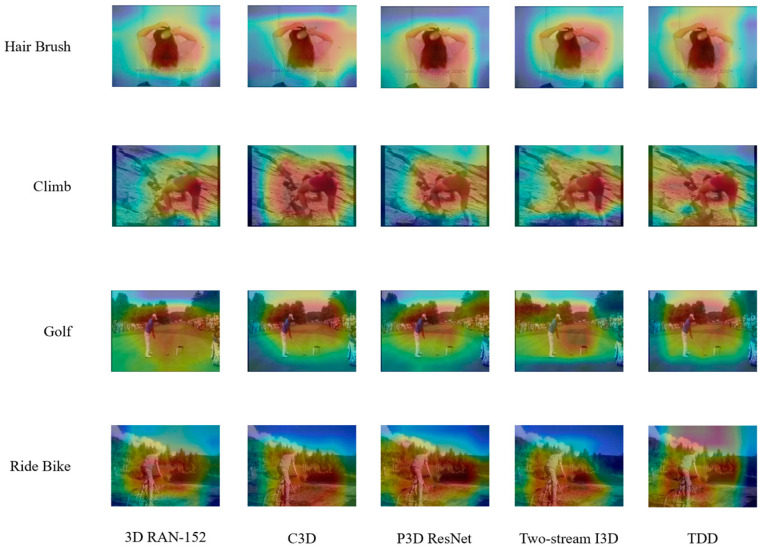
Visual comparison of contrast experiments using HMDB51.

Similarly, to thoroughly assess and validate the efficacy and distinctiveness of our proposed model, we performed a comprehensive visual comparison of the highly regarded and advanced models within the relevant domain. As shown in Figure 7, the specific videos and pictures that were carefully chosen for this comparative analysis were deliberately designed to be perfectly aligned and identical to those utilized in the visualization process of the ablation experiments. This meticulous selection and consistency of materials were primarily designed to streamline and enhance the convenience and accuracy of the comparison process.

When we meticulously and methodically contrasted the visual outcomes and manifestations of our model and advanced contrast models, it was evident that the model proposed and developed in this study showed notably better performance across diverse aspects and evaluation criteria. This enhanced performance is visually discernible and statistically significant. Moreover, integrating this visual comparison with the corresponding detailed comparison data, which covered a wide array of quantitative metrics and performance indicators, provided even more compelling and conclusive proof of the model’s undeniable superiority. This clearly demonstrates that our model has unique qualities and capabilities, allowing it to outperform and surpass current relatively advanced contrast models and firmly establish its position as a highly promising and competitive solution for handling the complex challenges and requirements of a specific task or field under study.

## 5. Conclusions

In this study, we designed a 3D residual attention network (3DRFNet) by seamlessly integrating a Channel Focus Enhancement Module (CFEM) and spatial attention mechanisms, thereby significantly enhancing the capture of spatiotemporal information. Of particular significance is the design of a cross-entropy loss function for gradient backpropagation, which effectively improves the convergence efficiency of the model. Moreover, the integration of Fast Fourier Convolution (FFC) further enhances the network’s ability to discern complex spatial-temporal dynamics. Our extensive experiments demonstrate that 3DRFNet outperforms other state-of-the-art methods on the UCF-101 and HMDB-51 datasets. Our network intelligently identifies the elements to emphasize or de-emphasize by reallocating weights to each channel and pixel in the intermediate feature maps, thereby highlighting crucial details. This mechanism significantly bolsters the self-representation and overall performance of a network. The outstanding performance of 3DRFNet, particularly its high accuracy rates of 98.7% on UCF-101 and 91.7% on HMDB-51, underscores its robustness and efficacy in capturing key behavioral features in videos. Looking ahead, we plan to extend the application of our networks to a broader range of video-related tasks with the goal of achieving equally remarkable results and making further contributions to the field.

## Figures and Tables

**Table 1:** Overview of action recognition datasets.

Dataset Name	Release Year	Number of Videos	Number of Action Classes	Average Video Length	Resolution
UCF101	2012	13,320	101	6.98 s	320 × 240
HMDB51	2011	6849	51	5.06 s	320 × 240
Kinetics	2017	650,000	400/600/700	0.86 s	Various

**Table 2:** This table shows the action recognition accuracy rates (%) for various attention submodule arrangements on the HMDB51 and UCF101 validation sets.

Combination	Channel	Spatial	FFC	UCF101 Accuracy (%)	HMDB51 Accuracy (%)
None				83.2	52.8
channel	✓			84.6	57.4
	✓		✓	90.1	70.89
spatial		✓	✓	92.6	78.8
		✓		85.2	59.6
**3DRFNet**	✓	✓	✓	**98.7**	**91.7**

**Table 3:** The performance of 3DRFNet using different loss functions on the UCF-101 and HMDB-51 datasets.

Loss Function	UCF-101 Acc (%)	HMDB-51 Acc (%)
Focal Loss	85.8	63.5
Dice Loss	85.5	63.2
Triplet Loss	85.7	63.4
Label Smoothing Loss	86.1	64
**Softmax Cross-Entropy**	**98.7**	**91.7**

**Table 4:** Comparison of action recognition accuracy rates (%) against current leading techniques in HMDB-51 and UCF-101 data collection.

Method	Dim	Pre-Trained	UCF-101 Acc (%)	HMDB-51 Acc (%)	Inference Time (ms)
C3D [45]	3D	Kinetics	85.4	63.3	120
P3D ResNet [46]	3D	Kinetics	85.3	63.5	110
Two-stream I3D [47]	3D	Kinetics	84.6	64.3	130
TDD [48]	2D	ImageNet	84.9	63.1	90
TimeSformer [49]	3D	Kinetics	87.2	65.1	150
Vision Vision Transformer [50]	3D	Kinetics	86.8	64.7	140
Audio-Visual SlowFast [51]	3D	Kinetics	87.0	65.0	160
Cross-Attention [52]	3D	Kinetics	86.6	64.5	135
MobileNetV3 + 3D CNN [53]	3D	Kinetics	85.8	63.6	105
**3DRFNet**	3D	Kinetics	**98.7**	**91.7**	91

## Data Availability

Data are contained within the article.
